# Healthcare workers' participation in a healthy-lifestyle-promotion project in western Sweden

**DOI:** 10.1186/1471-2458-11-448

**Published:** 2011-06-08

**Authors:** Ingibjörg H Jonsdottir, Mats Börjesson, Gunnar Ahlborg

**Affiliations:** 1 The Institute of Stress Medicine, Carl Skottsbergs gatan 22B, SE 413 19 Gothenburg, Sweden; 2 Department of Physiology, Institute of Neuroscience and Physiology, Sahlgrenska Academy at the University of Gothenburg, Gothenburg, Sweden; 3 Department of Emergency and Cardiovascular Medicine, Institute of Medicine, Sahlgrenska Academy at the University of Gothenburg, Gothenburg, Sweden; 4 Department of Public Health and Community Medicine, Institute of Medicine, Sahlgrenska Academy at the University of Gothenburg, Sweden

## Abstract

**Background:**

Healthcare professionals play a central role in health promotion and lifestyle information towards patients as well as towards the general population, and it has been shown that own lifestyle habits can influence attitudes and counselling practice towards patients. The purpose of this study was to explore the participation of healthcare workers (HCWs) in a worksite health promotion (WHP) programme. We also aimed to find out whether HCWs with poorer lifestyle-related health engage in health-promotion activities to a larger extent than employees reporting healthier lifestyles.

**Method:**

A biennial questionnaire survey was used in this study, and it was originally posted to employees in the public healthcare sector in western Sweden, one year before the onset of the WHP programme. The response rate was 61% (n = 3207). In the four-year follow-up, a question regarding participation in a three-year-long WHP programme was included, and those responding to this question were included in the final analysis (n = 1859). The WHP programme used a broad all-inclusive approach, relying on the individual's decision to participate in activities related to four different themes: physical activity, nutrition, sleep, and happiness/enjoyment.

**Results:**

The participation rate was around 21%, the most popular theme being physical activity. Indicators of lifestyle-related health/behaviour for each theme were used, and regression analysis showed that individuals who were sedentary prior to the programme were less likely to participate in the programme's physical activities than the more active individuals. Participation in the other three themes was not significantly predicted by the indicators of the lifestyle-related health, (body mass index, sleep disturbances, or depressive mood).

**Conclusion:**

Our results indicate that HCWs are not more prone to participate in WHP programmes compared to what has been reported for other working populations, and despite a supposedly good knowledge of health-related issues, HCWs reporting relatively unfavourable lifestyles are not more motivated to participate. As HCWs are key actors in promoting healthy lifestyles to other groups (such as patients), it is of utmost importance to find strategies to engage this professional group in activities that promote their own health.

## Background

Lifestyle-related diseases are increasing, and today non-communicable diseases such as cardiovascular illnesses, cancer, and diabetes are reaching epidemic proportions worldwide [1]. Successful strategies to promote healthier lifestyles, including physical activity (PA) and diet, are thus of great importance, and the workplace has been identified as a promising setting for health promotion [1,2]. Indeed, many worksite health promotion (WHP) programmes are considered a feasible way to reach many individuals, and they are shown to positively affect health and increase employees' productivity [3-5]. The largest benefits however are most likely reached if individuals with the poorest health participate in the programme, but this varies greatly across studies [2]. Participation in WHP programmes seems to be a complex process as, for example, the same health-risk behaviour can be differently related to participation depending on the type of programme offered [6,7]. For example, obese individuals were less likely to participate in an on-site fitness programme than low-risk individuals, while the obese risk group was more likely to participate in a wellness educational programme [7].

Healthcare workers (HCWs) should theoretically have the necessary education and environment to adopt a healthy lifestyle, and they supposedly also should have a higher participation rate in WHP programmes. HCWs are, for several reasons, considered to be a key group in health promotion, especially due to the fact that the healthcare system reaches a substantial number of people in need of lifestyle changes such as increased PA [8]. Furthermore, healthcare professionals are considered to be the most credible source of health information [9]. HCWs' lifestyles can play an important role in increasing awareness among patients regarding lifestyle changes, because HCWs' own lifestyle habits and interests in lifestyle behaviour have been shown to positively influence their counselling practices and attitudes [9-11]. The international movement 'Health Promoting Hospitals and Health Services', which was initiated by the World Health Organization (WHO), highlights the importance of also focusing on the health and lifestyle of the employees. HCWs have, in several studies, been shown to report healthier lifestyles than the general population [12-14] but all studies do not support the notion that HCWs always report healthier lifestyle-related behaviour regarding, for example, smoking, PA, or dietary habits compared to other individuals [15,16]. Few studies are available on the participation rate of HCWs in WHP programmes. Stein and co-workers showed that hospital workers' participation in at least one health-promotion activity was slightly below 30% [17], while McCarty and Scheuer showed that employees in healthcare organizations in the United States had a participation rate of 20% and 9%, respectively, in two separate fitness programmes offered to the employees [18]. Compared with the general participation levels in WHP programmes, ranging between 10% and 64% with a median of 33%, it does not seem that HCWs are more prone to participate in WHP programmes than other working populations [2].

In 2005, a three-year-long project promoting healthy lifestyles was initiated in western Sweden as a joint venture between a large Swedish manufacturing corporation and the public healthcare provider in western Sweden. Here we explore participation in this WHP programme among the employees working in the public healthcare sector by using data from a biennial survey that started in 2004. The aim was also to find out whether HCWs with baseline characteristics indicating poorer lifestyle-related health did take part in the activities to a larger extent than employees without these characteristics.

## Methods

### Lifestyle in the West project

The Lifestyle in the West (LIW) project was initiated as a joint venture between two of the largest employers in western Sweden: a manufacturing corporation representing the private sector and the regional public healthcare organization. The aim of the project was to promote healthy lifestyle behaviour among the employees. The target population for the project was in total the 65,000 employees of these two organizations. This study, however, considers only employees working in the healthcare sector. This 3.5-year WHP project, conducted between 2005 and 2008, used a broad all-inclusive approach, relying on individuals' decisions to participate instead of targeting specific at-risk individuals. The key phrases of the LIW project were: focus on the healthy (not on disease), achievement of balance (between work and leisure, and between activity and recovery), no pointing fingers (persons at risk were not specifically targeted), and individual will (all initiatives of the project were available for the individual, but not mandatory). Four lifestyle-related themes were decided on: physical activity, nutrition, sleep, and happiness/enjoyment. During a period of six to nine months, special attention was given to each theme, starting with the physical activity theme in 2005. A group of experts were appointed for each theme, responsible for initial training of 150-200 employees, of both sexes with different ages and occupations, to become local 'health coaches'. During the duration of the project, the health coaches were continuously updated, and provided with educational material (a website, PowerPoint presentations, and books) for each theme. Chief executives and union representatives also received training with the clear aim of increasing awareness on the importance of promoting a healthy lifestyle and lifestyle changes among the employees and making it possible for the employees to participate in different activities, even during working hours.

During the duration of the project, the health coaches functioned as local practical ambassadors of the project, organizing seminars, competitions, and theme-related events. Ideas for activities were provided in a project book available for the health coaches, but the activities could differ considerably between different workplaces. Educational books for each theme were produced and distributed to all employees. Examples of general activities were a pedometers competition between different departments, a cookbook with healthy recipes, environmental certification of hospital food and restaurants, and production of an educational TV show on healthy lifestyles. Furthermore, thousands of local activities were initiated during the project period.

### Participants

At the time of the study, employees of the public healthcare organization numbered around 48,600 (80% women). Approximately one year before the onset of the LIW project, a questionnaire survey was posted to a random sample of 5300 employees. The sample included mainly HCWs (around 80%), others being administrative personnel working in hospitals, primary care, dental care, or central administration. An inclusion criterion was having been employed for at least one year and working 50% or more of full-time. A total of 3207 individuals (response rate 61%) consented to participate and completed the questionnaire (Table 1). Seventy-three percent were working in hospitals (one large university hospital, four medium sized and eight small hospitals), 13% in primary care, 8% in dental care, and 5% in central administration offices. When comparing responders with the non-responders, women tended to respond more frequently than men and middle aged persons were slightly overrepresented (the proportion of women was 1.7% higher and the age group 45-54 years 1.3% higher compared to the random sample population). The responders of the baseline survey in 2004 were asked to participate in follow-ups to be conducted every second year. The response rate at the first follow-up in 2006 was 85%. At the second follow-up in 2008, 1971 persons responded (83%), of whom 1859 (94%) answered the question of whether they had participated in any LIW activity during the duration of the project. The study was approved by The Regional Ethical Review Board in Gothenburg and conduced in compliance with the Helsinki declaration.

**Table 1:** Characteristics of all the healthcare workers responding at baseline (n = 3207).

Variable (n)	Classification	Characteristics n (%)
Sex (3207)	Women	2772 (86)
	Men	435 (14)
		
Age (3207)	Years	
	18-34	461 (14)
	35-44	801 (25)
	45-54	1104 (34)
	55+	841 (26)
		
Educational level^1 ^(2900)	Lower	1160 (40)
	Higher	1740 (60)
		
BMI (3166)	Mean (SD)	24.5 (SD 3.6)
		
- Underweight	BMI< 18.5	31 (1)
- Normal weight	BMI 18.5-24.9	1901 (59)
- Overweight	BMI 25-29.9	1011 (32)
- Obese	BMI > 30	223 (7)
		
Physical activity (3185)	Sedentary	470 (15)
	Light activity	1697 (53)
	Moderate to vigorous	1018 (32)
		
Sleep disturbances (3188)	No	2346 (73)
	Yes	842 (26)
		
HAD-D score (3143)	< 7	2584 (82)
	≥ 7	559 (18)
		
Expectation/feelings about going to work (3164)	Happy or fairly happy and satisfied at the thought of an interesting workday ahead	2447 (77)
	Neither positive nor negative feelings about going to work	431 (14)
	Rather or strong uneasy feeling about going to work	286 (9)
		
Self-reported health (n = 3,195)	Very good/good	2620 (82)
	Neither good or bad	388 (12)
	Not good	187 (6)

^1 ^Lower = high school/vocational; Higher = college/university. Missing are those responding to 'other professions' that could not be categorized into lower or higher education.

BMI = Body mass index.

HAD-D = Subscale depression of the Hospital Anxiety and Depression Scale. A score of ≥ 7 is used to indicate a relative lack of joy/happiness.

### The survey

The general aim of the survey was to investigate different aspects of psychosocial work environment, stress, and stress-related health in the target population, including a range of demographic factors and lifestyle parameters. At the four-year follow-up in 2008, questions about the LIW project were included, and the participants were asked to respond 'yes', 'no', or 'do not know' to the question of whether they had participated in any LIW activity. A following question was 'If yes, which theme(s) (nutrition, sleep, physical activity, and/or happiness/enjoyment) did you participate in?' Among the variables available from the baseline data, four indicators of lifestyle-related health/behaviour were identified that seemed best related to the respective theme of the LIW project. Thus, body mass index (BMI) was considered to best relate to the nutrition theme, self-reported PA to the physical activity theme, sleep disturbances to the sleep theme, and general mood (depressive or not) and feelings about work (positive or not) to the happiness/enjoyment theme. A single item on self-rated general health was also used as an indicator of a variety of experienced health problems.

#### Self-reported physical activity

The participants rated their PA level according to an adapted version of the widely used four-level scale originally developed by Saltin and Grimby [19]. This simple instrument has been shown to discriminate between sedentary and active counterparts regarding maximal oxygen uptake [20] and has been validated against biological measures [21]. Furthermore, it has been widely used in several large epidemiological studies that show the relation between self-reported PA and morbidity as well as to mortality [22-24].

The participants reported the level that best corresponded to their PA during the last three months: mostly sedentary (group 1), light PA (such as gardening, or walking or bicycling to work) at least two hours a week (group 2), moderate PA (such as doing aerobics, dancing, swimming, playing football, or doing heavy gardening) at least two hours a week (group 3), or vigorous PA several times a week, for at least five hours with high intensity (group 4).

Due to the fact that only 2.4% of the participants reported vigorous PA, we reduced the four categories to three distinctive groups: sedentary (group 1), light PA (group 2), and moderate-to-vigorous PA (groups 3 and 4). When dichotomized in two categories (sedentary and physically active), both the light PA and moderate-to-vigorous PA groups were placed together as the physically active group.

#### Sleep disturbance

Two items were used to construct the sleep-disturbance variable. The participants were asked to answer how often during the past three months they had experienced: a) difficulty falling asleep and b) repeated awakenings with difficulty falling back asleep. Five different response alternatives were used for both items as follows: 1) never, 2) seldom, 3) sometimes, 4) several times a week, or 5) every day. Participants who selected alternative four or five for at least one of the two items were defined as suffering from a sleep disturbance.

#### Mood

The Hospital Anxiety and Depression (HAD) scale was included in the questionnaire [25] and the subscale for depression (HAD-D) was used as an indication of presence or absence of joy/happiness at baseline. This subscale has seven items, each with four response categories. A total sum score of seven or more was defined as a relative lack of joy/happiness (depressive mood).

#### Feelings about work

A single question--'What feelings do you usually have about your work when you are on your way there?'--was used to gauge how respondents felt about their jobs. The five different response alternatives are as follows: 1) I feel happy and satisfied about the interesting workday ahead, 2) I have a fairly positive feeling about work, 3) I have neither positive nor negative feelings about work, 4) I feel rather uneasy about work, and 5) I have a strong uneasy feeling about work. These five alternatives were reduced to three categories as alternatives 1 and 2 were combined into the category 'Happy or fairly happy and satisfied about the workday ahead' and similarly alternatives 4 and 5 were put into the same category of 'Rather or strong uneasy feeling about going to work', leaving alternative 3 as 'Neither positive nor negative feelings about going to work'.

#### Self-rated general health

The single item taken from the SF-36 short-form health survey was used: 'In general, how would you describe your health?' [26]. From the five response alternatives, three categories of self-rated health were defined: very good (excellent/very good), neither good nor bad (neutral), and not good (fair/poor).

### Procedures and analysis of dropouts

The LIW project started in May-June 2005 and continued until May 2008. Characteristics of the responders at baseline in May-June 2004 were analysed to explore indicators of lifestyle-related health/behaviour in the total population before the onset of the healthy-lifestyle-promotion project. Data from the four-year follow-up, conducted in May 2008, was then used to analyse how participation in LIW was related to these indicators, among the 1859 HCWs available for analysis. When comparing dropouts with the individuals still responding at follow-up, the latter population was slightly younger on average, 46.4 years (SD 9.3) compared to 47.4 years (SD 11.1) for the dropouts (p = .001). This was expected since individuals who retired from work between 2004 and 2006 were not invited to participate in 2008. The proportion of women was greater in the responding population 2008 (62.4% versus 55.2%; p = 0.004). The distribution according to educational level, based on profession (whether the occupation requires college/university studies), was slightly different between the groups as 61% of the responders were classified as having higher education compared to 58% among the dropouts (p = .049). BMI was similar in the two groups, but the responders seem to constitute a slightly healthier population as only 4.5% reported their general health to be 'not good', compared to 8% among the dropouts (p = .0005). Furthermore, depressive mood (HAD-D score ≥ 7) was found in 16% and 21%, respectively (p = .001). Also, the proportion experiencing sleep disturbances at baseline was lower in the former group (37.3% versus 41.6%; p = 0.031), and the proportion reporting a sedentary lifestyle was 13.6% compared to 16.6% in the dropout group (p = .024).

### Statistics

Data from the survey in 2004 was used to analyse and describe the target population for the LIW project. No data imputation was done, and the number of observations can thus vary somewhat for the respective analysis. Descriptive statistics are given in terms of counts and percentages. Pearson's chi-square test was used to analyse group differences except for BMI and age where Mann-Whitney U test was used. The level of significance was set at p < 0.05. The association between the respective indicator of lifestyle-related health/behaviour and participation was assessed by Cox regression and expressed as participation ratios (PR) (which equals prevalence ratios at follow-up) with 95% confidence intervals. All analyses were performed by using SPSS version 15.0.

## Results

### Baseline characteristics of the target population

Thirty-nine percent of the responders were categorized as being overweight or obese, based on BMI (Table 1). The sedentary PA category was affirmed by 15%, and 26% reported sleep disturbances. According to the HAD scores, a relative lack of joy/happiness was observed in 18%. A large majority reported a happy or rather positive feeling about going to work, while 9% reported that they had a 'rather or strong' uneasy feeling about work. Furthermore, the majority of the population rated their health as good or very good according to the SF-36 self-rated health question (Table 1).

### Characteristics of participants and non-participants in the LIW project

The participation rate in the LIW project was 21.5% as 400 out of 1859 responded that they participated in any LIW activity. Among those responding 'Yes' to participation, 319 (80%) reported that they participated in the physical activity theme, 124 (31%) participated in the nutrition theme, 107 (27%) in the enjoyment/happiness theme, and 99 (25%) in the sleep theme. Ninety-five of the 319 individuals participating in the physical activity theme also took part in the nutrition theme and 76 in the sleep theme. Fifty-four participants (14%) reported that they participated in all four themes.

The characteristics of the participants and non-participants were analysed by using baseline data measured before the onset of the LIW project. Participation was more common among women (23%) compared to men (11%) (p < .0005). The participants were somewhat older than the non-participants (mean age 47.8; SD 8.1 compared to 45.8; SD 9.6; p = .001), but no difference was seen between the groups regarding BMI (mean 24.1; SD 3.2 and 24.6; SD 3.7, respectively; p = .201). The group that participated in any LIW activity reported a generally more positive health-related lifestyle, because fewer individuals in this group reported sleep disturbances and sedentary physical behaviour (Table 2). Also the participants had generally more positive feelings about work, but the proportion reporting a depressive mood or poor general health was similar in the two groups. The educational level did not differ significantly between participants and non-participants.

**Table 2:** Comparison of health-related characteristics between participants and non-participants measured before the onset of the lifestyle project

	Participants (n = 400) % (n)	Non-participants (n = 1459) % (n)	p-value
			
BMI			
- < 18.5	1 (3)	1 (16)	.168
- 18.5-24.9	64 (254)	61 (874)	
- 25-29.9	30 (118)	31 (441)	
- > 30	5 (20)	8 (113)	
			
Physical activity			
- Sedentary	8 (30)	16 (227)	.0005
- Physically active	93 (370)	84 (1226)	
			
Sleep disturbances			
- Yes	21 (82)	26 (381)	.025
- No	79 (314)	74 (1073)	
			
HAD-D score			
- < 7	86 (335)	83 (1189)	.159
- ≥ 7	14 (55)	17 (245)	
			
Feeling about going to work	84 (335)	79 (1133)	.008
- Positive	12 (48)	13 (185)	
- Neutral	4 (16)	8.5 (122)	
- Uneasy			
			
Self-reported health			
- Very good/good	87 (346)	83 (1208)	.064
- Neither good nor bad	11 (43)	12 (174)	
- Not good	3 (10)	5 (74)	
			
Educational level^1^			.435
- Lower	39 (140)	37 (496)	
- Higher	61 (216)	63 (842)	

Only persons replying to the question at the four year follow-up whether they had participated in the Lifestyle in the West project are included in the analysis (n = 1859)

BMI = Body mass index.

HAD-D = Subscale depression of the Hospital Anxiety and Depression Scale. A score of ≥ 7 is used to indicate a relative lack of joy/happiness.

^1 ^Lower = high school/vocational; Higher = college/university. Missing are those responding to 'other professions' that could not be categorized into lower or higher education.

### Participation in different themes

When calculating PR by Cox regression, it was found that women were more likely to have participated in the sleep and physical activity themes, and the estimates were similar for the nutrition theme, although it did not reach a significant difference (Table 3). The gender difference was even more pronounced for the happiness/enjoyment theme as only one man out of 107 participated. Sedentary individuals were less likely to participate in PA than those reporting any level of PA at baseline. Participation in other themes (nutrition, sleep, or happiness/enjoyment) was not significantly predicted by the item chosen to represent each theme (such as BMI for nutrition) (Table 3). Workers with neutral feelings about work tended to be more likely to participate in the happiness/enjoyment theme than those reporting happy feeling about work (2.63; CI 0.97-7.15). Uneasy feelings about work did not predict participation.

**Table 3:** Unadjusted and adjusted participation ratios (PR) with 95% confidence intervals (CI) for sex and the respective baseline indicator relevant for each of the different themes, as analysed by Cox regression

Theme (number who participated)	Category	Unadjusted PR (95% CI)	Adjusted PR (95% CI)
**Nutrition **(124)			
Sex	Men	1	1
	Women	2.02 (0.99-4.13)	1.90 (0.82-4.37)^1^
BMI	Normal weight	1	1
	Overweight/obese	1.13 (0.79-1.62)	1.07 (0.71-1.59)^2^
			
**Sleep **(99)			
Sex	Men	1	1
	Women	2.62 (1.06-6.43)	3.24 (1.01-10.33)^1^
Sleep disturbances	No	1	1
	Yes	1.08 (0.69-1.70)	0.97 (0.62-1.52)^3^
			
**Physical activity **(319)			
Sex	Men	1	1
	Women	2.08 (1.32-3.27)	2.09 (1.25-3.48)^1^
Physical activity level	Physically active	1	1
	Sedentary	0.51 (0.33-0.77)	0.51 (0.34-0.77)^4^
			
**Happiness/enjoyment **(107)			
Sex	Men	NA	
	Women		
Depressive mood (HAD-D)	Low (< 7)	1	
	High (≥ 7)	0.66 (0.36-1.21)	

Only persons replying at the four year follow-up to the question whether they participated in the Lifestyle in the West project are included in the analyses (n = 1859)

^1 ^Adjusted for educational level and age.

^2 ^Adjusted for educational level, age, and sex.

^3 ^Adjusted for age and sex.

^4 ^Adjusted for age.

NA = Not statistically analysed as only one man out of 107 participated.

BMI = Body mass index.

HAD-D = Subscale depression of the Hospital Anxiety and Depression Scale.

## Discussion

The main result of the present study was that participation in this large non-targeted WHP programme aimed at HCWs was low, around 20%, indicating that HCWs are not more prone to participate in health-promotion activities compared to what has previously been observed in other populations [2]. Thus, our results are in line with the few studies previously published on this subject [17,18]; that is, HCWs do not seem to participate in WHP programmes to the extent that perhaps might be expected considering their knowledge on lifestyle-related health. In addition, we confirm previous studies showing that women have a higher participation rate than men, even among HCWs. Interestingly, our study shows that HCWs reporting risk indicators such as physical inactivity and sleep disturbances may, in fact, be less motivated to change their lifestyle in these respects, as sedentary workers were less likely to participate in the physical activity theme, and sleep disturbances or high BMI did not motivate respondents to participate in activities related to sleep or nutrition. It is noteworthy that participation was not related to educational level. The results thus indicate that, despite a supposedly good knowledge of health-related issues and higher awareness of the importance of lifestyle-related changes for health and well-being, HCWs reporting relatively unfavourable lifestyles/well-being are not reached by a non-targeted lifestyle WHP programme.

It can also be speculated that HCWs would initially report somewhat healthier lifestyles than the general population. The Swedish national public health survey shows that 12.9% of the women in western Sweden report a sedentary lifestyle [27] compared to 15% among the HCWs in our study. The self-rated question on PA was almost identical for these two surveys; however the age group differs considerably, as women aged 18-84 years participated in the national survey, which is a limitation when attempting to compare these two studies. No firm conclusion can be drawn as a direct statistical comparison between the groups could not be performed, but we can cautiously imply that it does not seem that HCWs differ much regarding physical activity behaviour compared to the general population.

Healthcare professionals play a central role in health promotion and lifestyle information towards patients as well as towards the general population, and it has been shown that healthcare professionals' own lifestyle habits can influence their attitude and counselling practice towards patients [9-11,28]. Thus, efforts to increase knowledge among healthcare professionals about their own lifestyle and identifying the potential barriers of lifestyle changes for this group, including motivation, could be central issues in the endeavour of promoting health among the general population.

One major concern of the public healthcare sector that might contribute to the relative low participation rate in the WHP programme is the demographic and organizational changes that have challenged this sector for many years. Increased workload, decreased employee control, and increased sickness absence among the employees have escalated since the 1990s [29]. At the same time, the WHO Collaborating Centre for Health Promotion in Hospitals and Health Care points out that even if the primary task of health care is to care about patients, the importance of the health of staff members should not be ignored. The health of the workers has to be taken into special consideration when making policy plans in an expert organization such as the healthcare sector. According to the results from our study, HCWs seem to have a similar need for health promotion as other working populations, and the challenge of reaching the subpopulations at risk seems to be the same. Previous research confirms that the most effective WHP programmes are those offering individualized risk-reduction counselling to the highest-risk individuals, and multi-component programmes seem to be most effective [5,30]. Hence, these kinds of programmes should be conducted even within the healthcare sector.

We are faced with several limitations regarding this study, primarily because the questions concerning participation in the WHP programme were incorporated in an ongoing survey that was available only to public-sector employees. Thus, no comparison can be made with the private-sector employees. The indicators of lifestyle-related health/behaviour had to be chosen among items already included in the original questionnaire survey, which was designed for another purpose. Ideally the indicators should have been tailored more specifically for the aim of the present study. However, the possibility to use data collected before the onset of the WHP project made it possible to prospectively assess the relation between the available indicators and participation, which can be regarded as a strength of the study. Due to the relatively small number of participants, it was not possible to assess whether factors such as working shifts or part-time, different areas of work (for example, hospitals or primary care), or different occupations had any influence on participation. Such questions should be addressed in future studies of determinants of participation in WHP programmes directed at HCWs.

## Conclusion

In conclusion, this study does not support the idea that HCWs are more prone to participate in WHP programmes compared to what has been reported for other working populations. They also report indicators of lifestyle-related health/behaviour in a similar manner as has been reported for the general population. Those with better self-reported health/lifestyles were more likely to participate than those with poorer indicators, who potentially would have benefitted most. As HCWs are key actors in promoting healthy lifestyles to other groups (such as patients), it is of utmost importance to find strategies to engage this professional group, also including those with less favourable lifestyles, in activities that promote their own health and healthy behaviour.

## Declaration of competing interests

The authors declare that they have no competing interests.

## Authors' contributions

All authors equally contributed to the planning of the analysis and interpretation of data. IHJ has conducted the statistical analysis and drafted the manuscript. MB has been actively working with the organization of the LIW project and made significant contributions to the writing of the paper. GA is responsible for the development and the design of the questionnaire used to collect the data and made significant contribution to the writing. All authors read and approved the final manuscript.

## Pre-publication history

The pre-publication history for this paper can be accessed here:

http://www.biomedcentral.com/1471-2458/11/448/prepub
